# Loss of vision as the first manifestation of amyloid light‐chain amyloidosis

**DOI:** 10.1002/jha2.845

**Published:** 2024-04-16

**Authors:** Niels W. C. J. van de Donk, Clément Huysentruyt, Mario R. P. Dhooge

**Affiliations:** ^1^ Department of Hematology Amsterdam UMC Vrije Universiteit Amsterdam Cancer Center Amsterdam Amsterdam The Netherlands; ^2^ Department of Pathology PAMM Eindhoven The Netherlands; ^3^ Department of Ophthalmology Elkerliek Ziekenhuis Helmond The Netherlands

**Keywords:** amyloidosis, diagnostic hematology, myeloma

1

A 70‐year‐old woman presented with slowly progressive loss of vision in both eyes over the course of 1.5 years. Ophthalmologic examination revealed multiple bilateral crystal‐like granular deposits in the cornea (Figure 1A), predominantly in the anterior stroma as well as in the conjunctiva of the right eye. In addition, prominent corneal nerves were observed bilaterally, caused by deposits along these nerves (Figure 1B).

**FIGURE 1:**
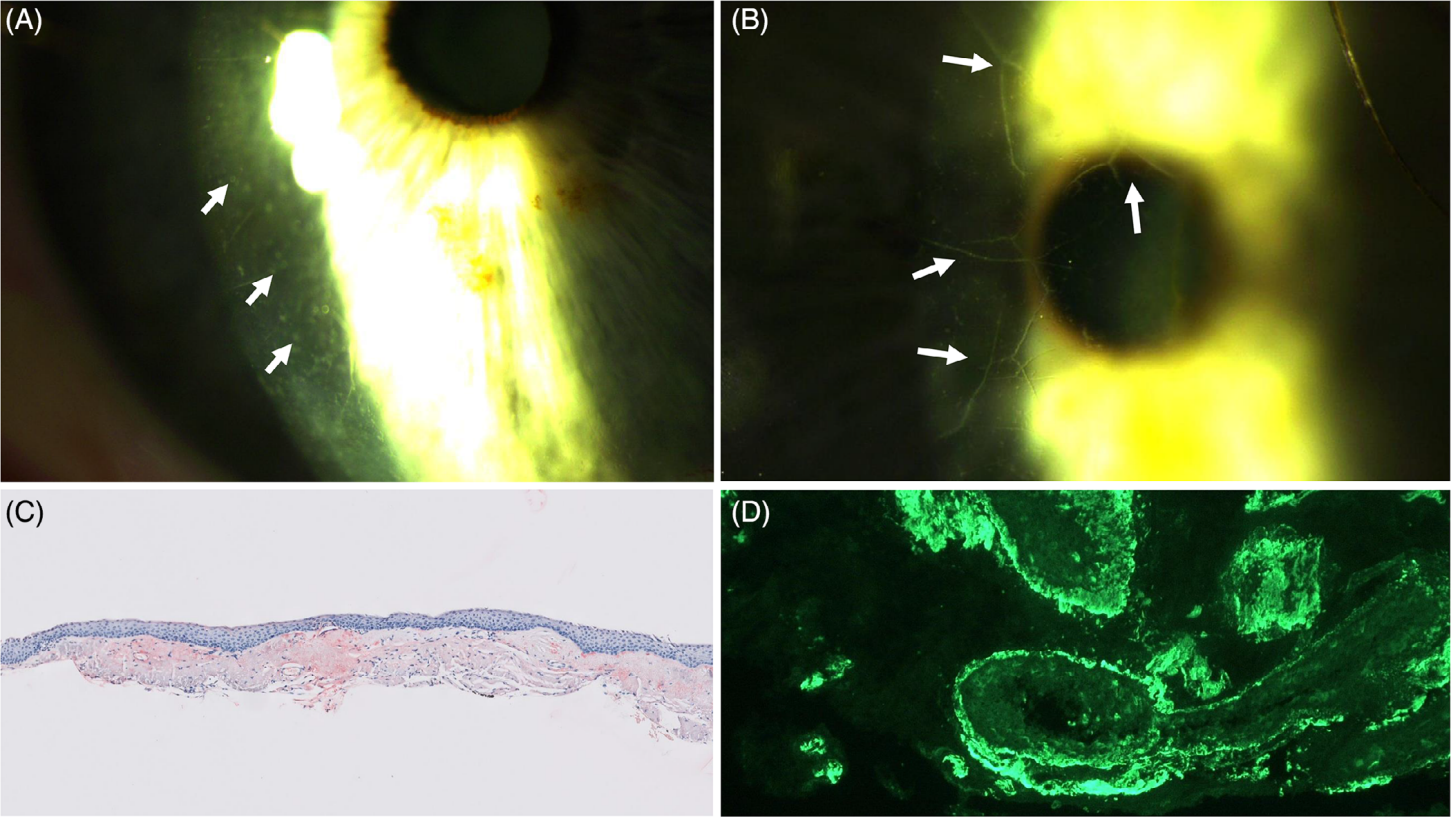
AL amyloidosis with ocular involvement. (A) Ophthalmologic examination showing multiple bilateral crystal‐like granular deposits in the cornea, predominantly in the anterior stroma. (B) Ophthalmologic examination demonstrating prominent corneal nerves. (C) Conjunctival biopsy showing red‐stained amyloid deposits on light‐microscopy (original magnification 50×). (D) Immunofluorescence staining showed that these deposits contained lambda light chains (original magnification 200×).

Amyloid was identified in a conjunctival biopsy by Congo red staining in combination with polarization microscopy (red‐stained deposits on light‐microscopy [Figure 1C; original magnification 50×]). Immunofluorescence staining showed that these deposits contained lambda light chains (Figure 1D; original magnification 200×) and were negative for kappa light chain or IgG. A diagnosis of corneal amyloid light‐chain (AL) amyloidosis was established.

Workup for AL amyloidosis included bone marrow analysis, which revealed the presence of 10% plasma cells. Laboratory findings included an IgG‐lambda M‐protein of 17 g/L with a moderate increase of lambda light‐chain level (lambda light chain: 63 mg/L; kappa light chain: 13 mg/L). Cytogenetic analysis of purified plasma cells revealed the presence of *t*(14;16) and gain1q21. Additional staging showed evidence of asymptomatic renal (proteinuria) and cardiac amyloidosis (typical MRI findings; Mayo 2004 stage II). There was no myeloma‐related organ damage (no acronym for hypercalcemia, renal failure, anemia, and bone disease (CRAB) manifestations). Therapy with bortezomib‐dexamethasone was initiated to eradicate the plasma cell clone, which is responsible for the production of toxic light chains, and 3 months later she achieved a complete hematologic response, which persists until now (2.5 years after starting therapy). Her visual impairment no longer deteriorated, and her proteinuria disappeared over time.

Corneal involvement is extremely rare in systemic AL amyloidosis and can lead to loss of visual acuity if not properly treated. In addition, it is noteworthy that, in this case, the ocular findings resulted in the eventual diagnosis of AL amyloidosis, before the development of advanced cardiac disease, which carries a very poor prognosis. The development of ocular pathology in our patient is probably related to specific physicochemical properties of the monoclonal lambda light chains produced by the clonal bone marrow‐localized plasma cells.

## AUTHOR CONTRIBUTIONS

N.v.d.D and M.D. treated the patient; C.H. performed the pathology evaluations; N.v.d.D. wrote the first version of the manuscript; M.D. and C.H. critically reviewed the manuscript.

## CONFLICT OF INTEREST STATEMENT

N.W.C.J.v.d.D. has received research support from Janssen Pharmaceuticals, AMGEN, Celgene, Novartis, Cellectis, and BMS and serves on advisory boards for Janssen Pharmaceuticals, AMGEN, Celgene, BMS, Takeda, Roche, Novartis, Bayer, Adaptive, Pfizer, Abbvie, and Servier, all paid to the institution. C.H. and M.D. declare no conflicts of interest.

## ETHICS STATEMENT

The authors have confirmed ethical approval statement is not needed for this submission.

## PATIENT CONSENT STATEMENT

The patient has given permission to anonymously describe this patient's case.

## Data Availability

The data are not publicly available because of privacy and ethical restrictions.

